# Structural determinant of BST-2-mediated regulation of breast cancer cell motility: a role for cytoplasmic tail tyrosine residues

**DOI:** 10.18632/oncotarget.22753

**Published:** 2017-11-29

**Authors:** Wasifa Naushad, Wadie D. Mahauad-Fernandez, Chioma M. Okeoma

**Affiliations:** ^1^ Department of Microbiology and Immunology, Carver College of Medicine, University of Iowa, Iowa City, IA, USA; ^2^ Department of Pharmacology, Stony Brook University, Stony Brook, NY, USA; ^3^ Atta-ur-Rahman School of Applied Bio sciences, National University of Sciences and Technology, Islamabad, Pakistan; ^4^ Division of Oncology, Departments of Medicine and Pathology, Stanford University School of Medicine, Stanford, CA, USA

**Keywords:** BST-2, tetherin, restriction factors, migration, invasion

## Abstract

There is now irrefutable evidence that overexpression of the innate immunity protein―BST-2, in breast cancer cells is implicated in tumor growth and progression. The cellular mechanisms that control BST-2-mediated effect in tumor progression involve enhancement of cancer cell motility―migration/invasion. However, the distinct structural elements of BST-2 that mediate breast cancer cell motility remain unknown. Here, we used various motility assays and different variants of BST-2 to examine the cellular and structural mechanisms controlling BST-2-mediated cell motility. We show that BST-2 silencing in various cancer cell lines inhibits cell motility. Restoration of BST-2 expression using construct expressing wild type BST-2 rescues cell motility. Mutational analysis identifies the cytoplasmic tail of BST-2 as a novel regulator of cancer cell motility, because cell motility was significantly abrogated by substitution of the BST-2 cytoplasmic tail tyrosine residues to alanine residues. Furthermore, in a spheroid invasion model, BST-2-expressing tumor spheroids are highly invasive inside 3D Matrigel matrices. In this model, the spreading distance of BST-2-expressing spheroids was significantly higher than that of BST-2-suppressed spheroids. Collectively, our data reveal that i) BST-2-expressing breast cancer cells in spheroids are more motile than their BST-2-supressed counterparts; ii) BST-2 cytoplasmic tail regulates non-proteolytic (migration) and proteolytic (invasion) mechanisms of breast cancer cell motility; and iii) replacement of the tyrosine residues at positions 6 and 8 in the cytoplasmic tail of BST-2 with alanine residues inhibits cell motility.

## INTRODUCTION

Breast cancer is responsible for ∼450,000 deaths per year worldwide and over 40,000 in the US. Five distinct subtypes of breast cancer―including, Luminal A, Luminal B, HER2 enriched, basal, and claudin low subtypes are recognized to have clinical significance [1–3]. In all of these cancer subtypes, the level of the antiviral innate immunity factor BST-2 is elevated [4–9]. Elevated BST-2 expression regulates cancer cell behavior, such as increased adhesion, anchorage independent growth, survival, migration, and invasion [6, 10, 11]. Exploration of the molecular and structural basis of BST-2-mediated migration and invasion could improve our understanding of the contribution of BST-2 in shaping the intricate cellular processes involved in breast cancer progression.

BST-2 is a type II transmembrane multifunctional innate immunity protein. Structurally, BST-2 is composed of an N-terminal cytoplasmic tail (CT), a transmembrane domain (TM), a coiled-coiled extracellular domain (ECD), and a C-terminal glycosylphosphatidylinositol (GPI)-anchor [12] in that order. The cytoplasmic tail of BST-2 contains a highly conserved double tyrosine motif (6Y7×8Y) implicated in clathrin-dependent endocytosis of BST-2 and in nuclear factor κ-B (NF-κB) activation [13–16]. The TM domain and the GPI anchor are separated by residues that make up the extracellular domain [17–19]. The N-terminus of the extracellular domain contains three cysteine residues involved in the formation of covalent cysteine-linked dimers [20–23]. The cysteine residues are located at positions 53, 63, and 91 of the human BST-2 and at positions 58, 68, and 96 of the mouse BST-2 [17]. Both the CT and ECD of BST-2 have been implicated in functional inhibition of virus replication [24] and virus release [18, 22]. Recently, BST-2 dimerization mediated by the ECD cysteine residues has been shown to enhance cell to cell and cell to ECM interaction, as well as in promoting cancer cell survival through the disruption of the anoikis machinery [9].

How BST-2 promotes cell motility is unclear. However, the involvement of the cytoplasmic tail of BST-2 in cellular signaling and the association of the extracellular domain in cell to cell interaction and in virus release [13, 18, 22, 24] prompted us to test whether these different domains of BST-2 contribute to the migratory and invasive behavior of breast cancer cells. To do this, we overexpressed well-characterized variants of human BST-2 [9] in BST-2-suppressed breast cancer cell lines [6]. We found that substitution of BST-2 cytoplasmic tail tyrosine residues with alanine residues impair cancer cell motility and invasiveness. Overexpression of these BST-2 variants in cancer cells enabled us to identify the tyrosine residues in the BST-2 cytoplasmic tail as critical in mediating proteolytic and non-proteolytic mechanisms of breast cancer cell motility.

## RESULTS

### BST-2 broadly promotes cancer cell migration

**Figure 1:**
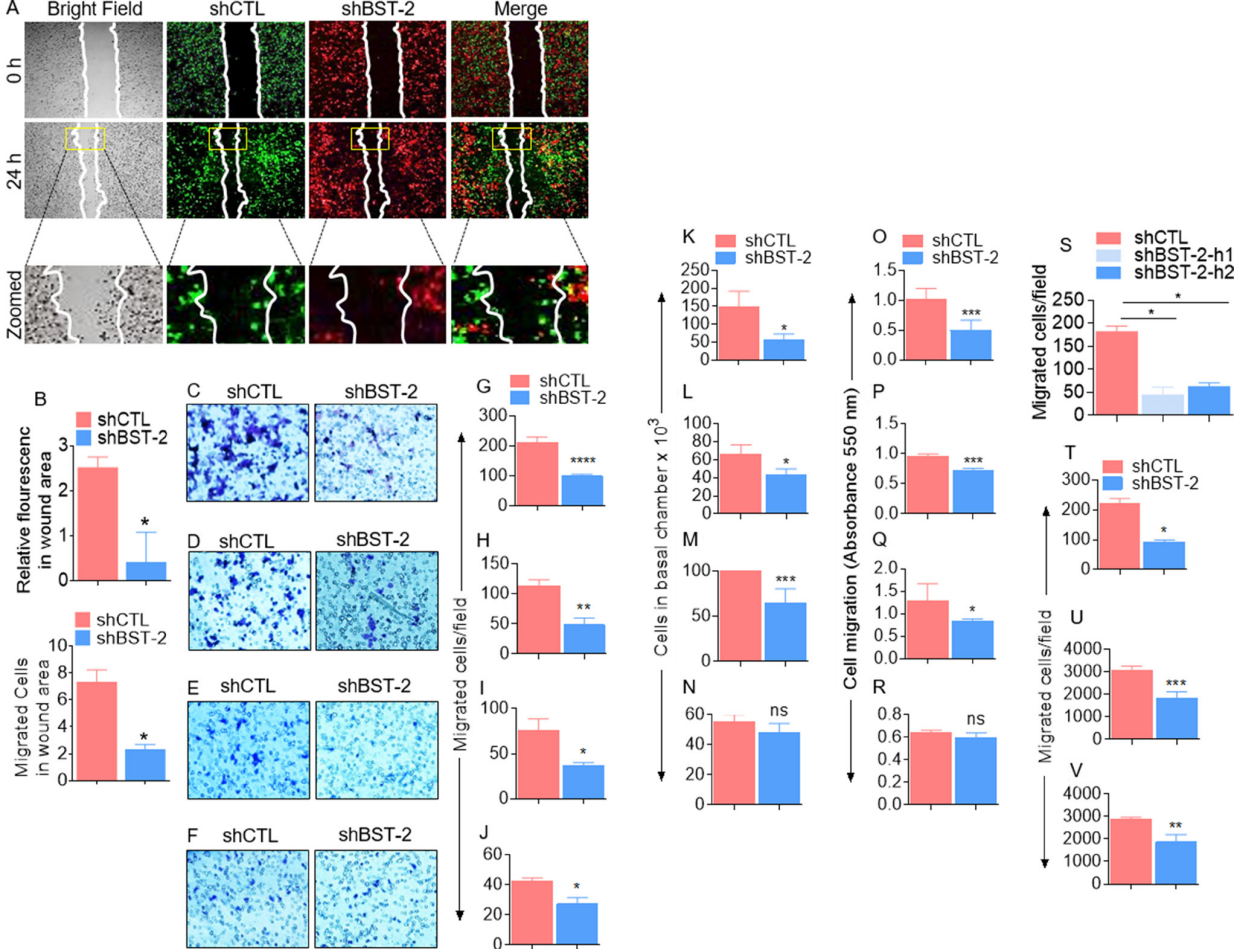
BST-2 broadly promotes cancer cell migration (**A**) Representative images of 4T1 shCTL (green) and shBST-2 (red) cells mixed 1:1 and co-cultured prior to wound healing assay performed at the indicated times. White lines on images depict initial wound border (0 h) and extent of wound closure (24 h). Area inside the yellow boxes is zoomed for each cell type in row 3. (**B**) Quantitation of relative mean fluorescence intensity (MFI, top) and cell numbers (bottom) that migrated into the wound area (yellow rectangle on images) at 24 h time point normalized to 0 h time point. (**C**–**R**) Representative experiments showing the effect of BST-2 on migration of isogenic 4T1, 4T07, 168FARN, 67NR shCTL and shBST-2 cell lines as analyzed by parallel assays including: (C–F) Microscopic imaging of Giemsa stained cells on the basal side of the trans well inserts. (G–J) Image J quantitation of trans well migration events. K-N. Hemocytometer-based enumeration of basal chamber cells. O-R. Spectrophotometric absorbance-based quantitation of migration events. (**S**) Migration rate of MDA-MB-231 shCTL, shBST-2-h1, and shBST-2-h2 cells. (**T**–**V**) Quantification of trans well migration of other non-breast cancer TZM-bl, SUP-T1, U937 cell lines expressing shCTL and shBST-2. For migration assays, cells from five different fields were blind-counted and values averaged. Error bars represent standard deviations. Significance was taken at *P* < 0.05^*^, *P* <0.01^**^, *P* < 0.001^***^, and *P* < 0.0001^****^. ns = not significant. Experiments were repeated more than three time with similar results.

Previous studies have demonstrated that BST-2 regulates cellular machinery that mediates migration and invasion of epithelia-derived breast cancer cells [6]. Here, we confirm that silencing BST-2 expression in the aggressive triple negative murine breast cancer cells 4T1 impairs cell motility in a two-dimensional (2D) co-culture wound healing assay (Figure 1A–1B). The number of BST-2-expressing (shCTL) breast cancer cells was higher in the wound area compared to BST-2-suppressed (shBST-2) cells within the same time (Figure 1A–1B). To further assess the role of BST-2 in cell migration, we used a three-dimensional (3D) trans well assay (Figure 1C) in which the basal chamber of the trans well is filled with medium containing FBS and fibronectin (FN) to compare the effect of reduced BST-2 expression in the migration of four isogenic mouse breast cancer cell lines—4T1, 4T07, 168FARN, and 67NR. Using three different readouts to compare the numbers of migrated shCTL versus shBST-2 cells, we show that silencing BST-2 expression (Supplementary Figure 1A–1C) in the aggressive 4T1 (Figure 1C, 1G, 1K, 1O) and 4T07 (Figure 1D, 1H, 1L, 1P) cells results in more significant decrease in cell migration compared to the less aggressive 168FARN cells (Figure 1E, 1I, 1M, 1Q). In contrast, loss of BST-2 in the non-aggressive 67NR cells had little or no significant effect on cell migration (Figure 1F, 1J, 1N, 1R). Similar observation was made with MDA-MB-231 human triple negative breast cancer cell line that contains high levels of endogenous BST-2 [6]. Following silencing of BST-2 expression with two different human BST-2-targeting shRNAs (Supplementary Figure 1D), we found that loss of BST-2 significantly reduces migration of MDA-MB-231 cells (Supplementary Figure 1).

The effect of BST-2 on cell migration is not limited to breast cancer cells because shRNA-mediated reduction of BST-2 level impairs the migratory potential of cells representative of other cancer types. These include cervical cancer (Figure 1T), T-cell lymphoblastic lymphoma (Figure 1U), and monocytic histiocytic lymphoma (Figure 1V) cells.

### BST-2 is a key factor in invasion of aggressive cancer cells

**Figure 2:**
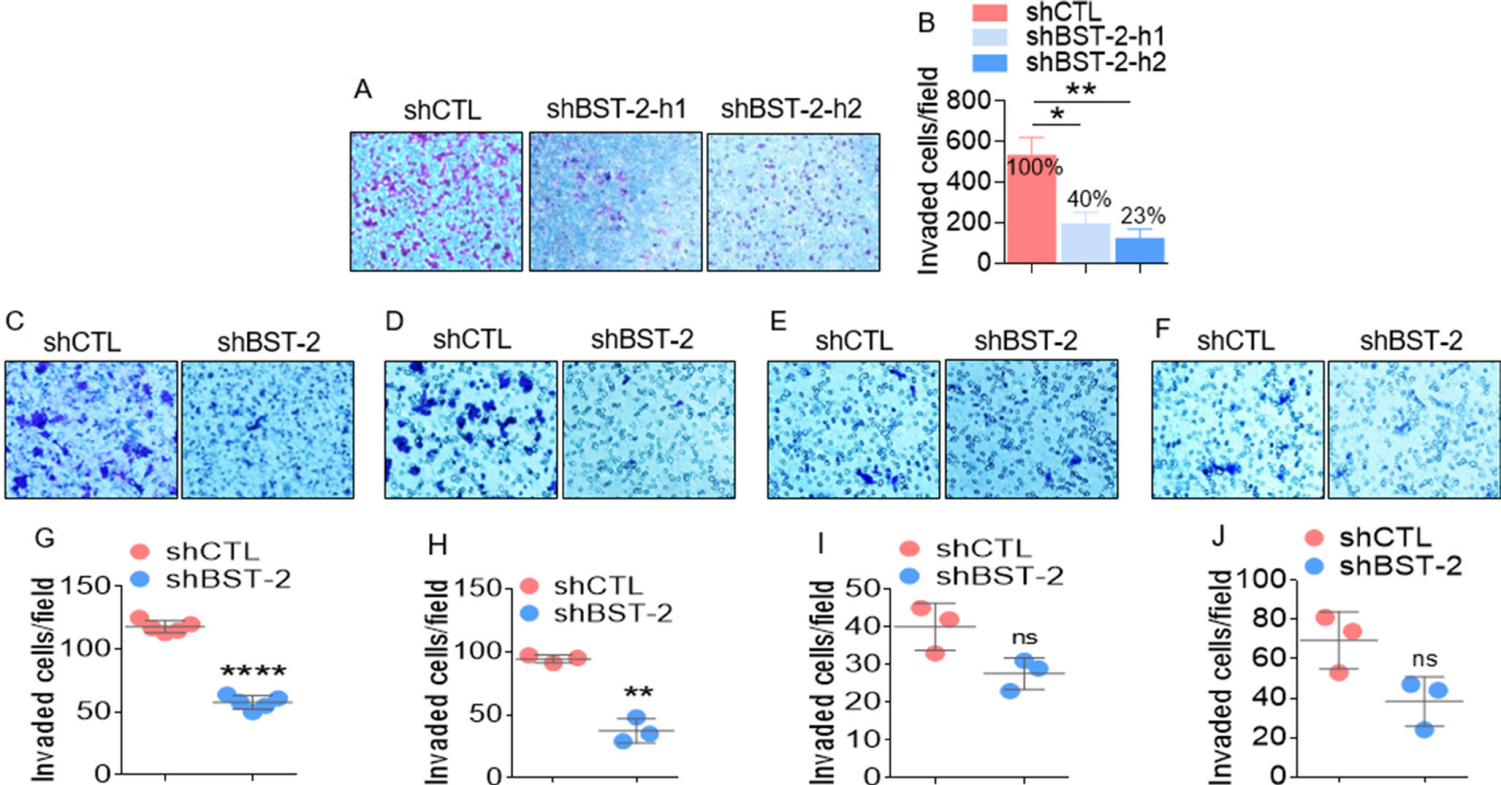
BST-2 broadly promotes invasion of cancer cells (**A**–**B**) Representative images and quantification of invasion rates of MDA-MB-231 shCTL, shBST-2-h1, and shBST-2-h2 cells through Matrigel-coated culture inserts. Numbers on graph in panel B represent % decrease in invasion. (**C**–**F**) Representative microscopic images of Giemsa-stained invaded isogenic 4T1, 4T07, 168FARN, 67NR shCTL and shBST-2 cells. (**G**–**J**) Image J quantitation of trans well invasion events shown in panels (C–F) In all experiments, cells from three to five different fields were blind-counted and values averaged or plotted as individual points. Error bars represent standard deviations. Significance was taken at *P* < 0.05^*^, *P* < 0.01^**^, and *P* < 0.0001^****^. ns = not significant. Experiments were repeated more than three time with similar results.

The effect of BST-2 on cell invasion is evident in the response of the aggressive human breast cancer cell line (MDA-MB-231) and four isogenic murine cancer cell lines. The invasiveness of MDA-MB-231 cells decreases from 100% in shCTL cells to 40% and 23% in shBST-2-h1 and shBST-2-h2 cells respectively (Figure 2A–2B). Additionally, BST-2 regulates invasion of the aggressive 4T1 and 4TO7 but not that of the weakly aggressive 168FARN and non-aggressive 67NR cells (Figure 2C–2F). The invasive capacity of the highly metastatic 4T1 cells reduces to 44% in shBST-2 cells compared to 100% in shCTL cells (Figure 2G). Similarly, the invasiveness of the moderately-metastatic 4TO7 decreased to 31.86% upon BST-2 silencing (Figure 2H). In stark contrast, silencing BST-2 expression had no significant effect on the invasion of the weakly-metastatic 168FARN (Figure 2I) and the non-metastatic 67NR isogenic cells (Figure 2J). Together, these data are consistent with previous reports that BST-2 promotes proteolytic cancer cell motility [6, 25].

### Breast cancer cells require BST-2 for efficient migration and invasion *in vitro*

**Figure 3:**
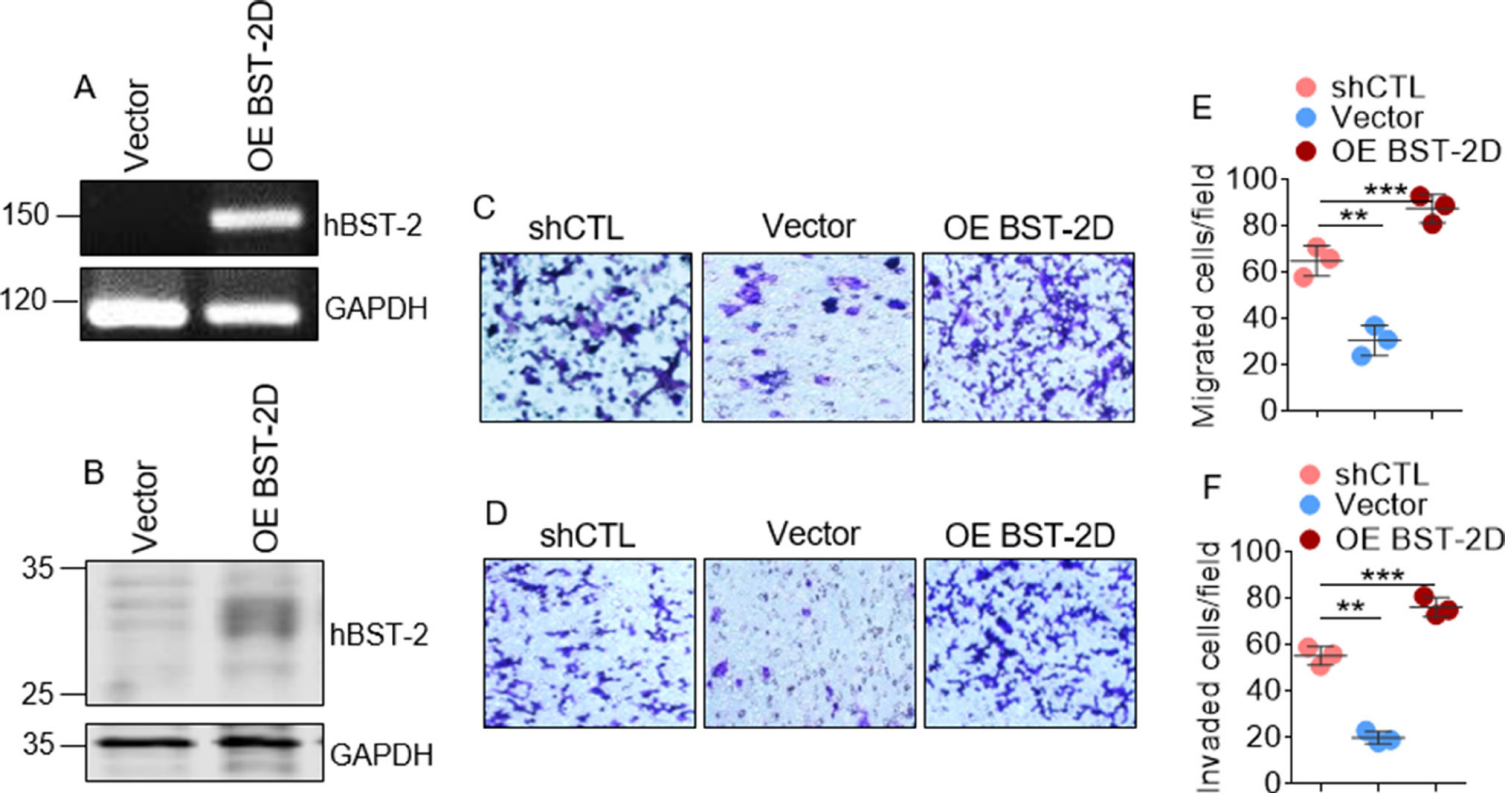
Exogenously expressed BST-2 rescues cancer cell migration and invasion in BST-2 suppressed cells (**A**) BST-2 mRNA and (**B**) BST-2 protein levels in BST-2-suppressed shBST-2 4T1 cells expressing empty vector (Vector) or overexpressing wild type human BST-2 (OE BST-2D). GAPDH was used as loading control in both cases. (**C**–**D**) Microscopic images of motility of shCTL-, Vector-, and OE BST-2D- expressing 4T1 cells assessed through trans-well migration and invasion assay respectively. (**E**–**F**) Quantification of rates of migration and invasion respectively of shCTL-, Vector-, and OE BST-2D- expressing 4T1 cells shown in panels (C–D) Numbers of migrated and invaded cells were calculated by blind-counting events from three different fields and plotted as individual points. Error bars represent standard deviations. Significance was taken at *P* < 0.01^**^ and *P* < 0.001^***^. Experiments were repeated more than three times with similar results.

To confirm that BST-2 regulates cellular machinery for migration and invasion, we used a previously reported cell line in which we rescued BST-2 expression in BST-2-suppressed 4T1 cells with wild type human BST-2 (OE BST-2D) that is resistant to shRNA against murine BST-2 [9]. Analysis of BST-2 mRNA (Figure 3A) and protein (Figure 3B) show that BST-2 expression was rescued in shBST-2 cells. Trans well migration and invasion studies show that exogenous expression of WT BST-2 rescues the migratory (Figure 3C and 3E) and invasive (Figure 3D and 3F) potential of BST-2-suppressed 4T1 cells. These data suggest that some elements of breast cancer cell motility require BST-2 to be present. However, the domain of BST-2 that controls cancer cell motility is unknown.

### Structure-function analysis reveals the requirement for BST-2 cytoplasmic tail for efficient breast cancer cell migration

**Figure 4:**
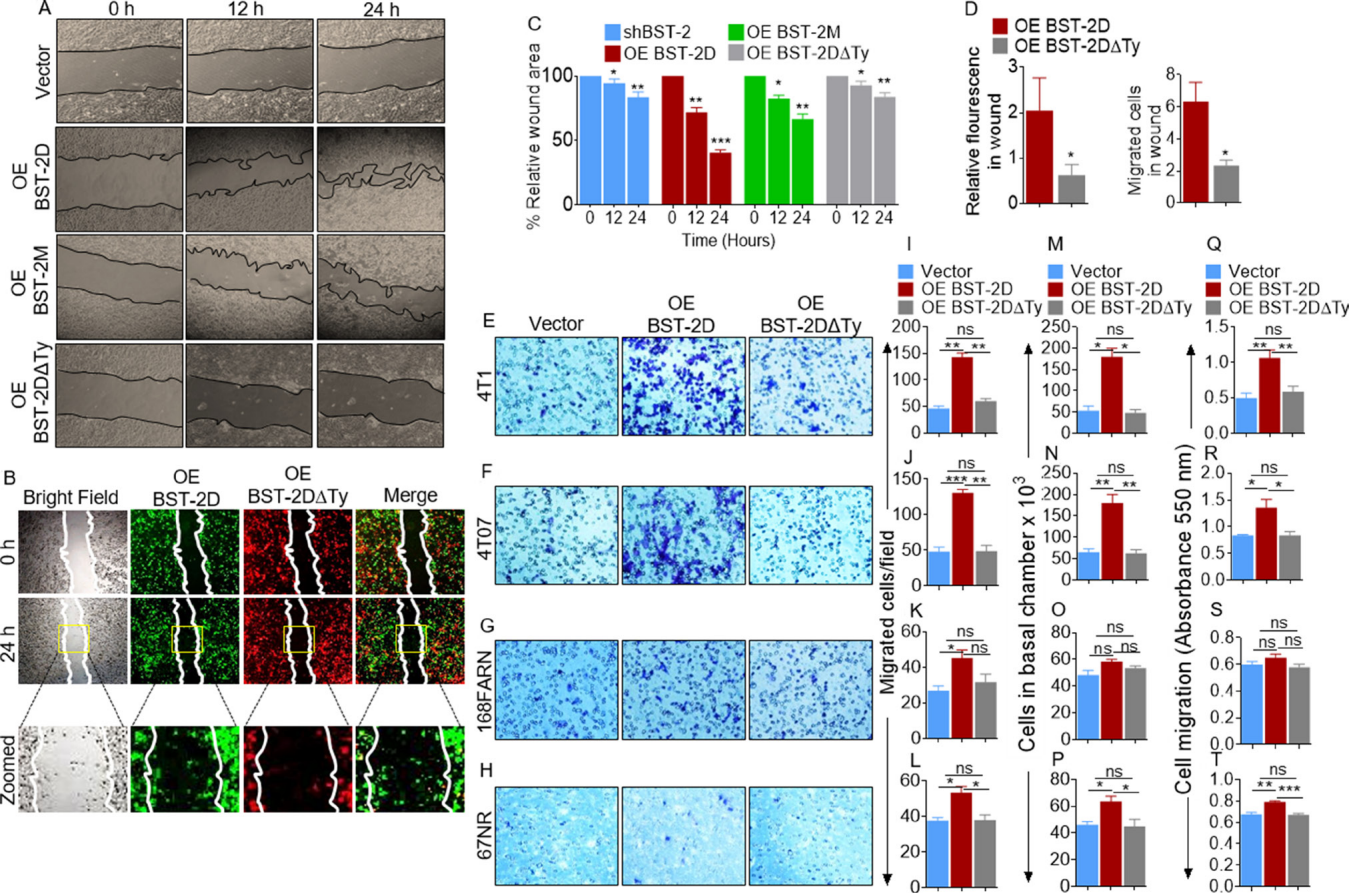
Identification of the domain of BST-2 that controls cell migration (**A**–**B**) Representative images of mono- and co- culture 2D migration of 4T1 cells expressing variants of BST-2 (shBST-2 (vector), OE BST-2D, OE BST-2M or OE BST-2DΔTy) at the indicated times using wound healing assay. In mono-culture migration, black lines on images depict wound border (0 h) and extent of wound closure (12–24 h). Co-culture migration at the indicated times used 1:1 cell mixture of OE BST-2D cells labelled green and OE BST-2DΔTy cells labelled red. White lines on images depict initial wound border (0 h) and extent of wound closure (24 h). Area inside the yellow boxes is zoomed (row 3) for each cell type. (**C**) Quantitation of rate of migration of cells in panel A analyzed as % wound closure. (**D**) Rate of migration of cells in panel B analyzed as relative florescence in wound area. (**E**–**T**) Representative experiments showing migration of isogenic 4T1, 4T07, 168FARN, 67NR shBST-2 cell lines expressing variants of BST-2 (shBST-2 (vector), OE BST-2D, OE BST-2M or OE BST-2DΔTy) analyzed by parallel assays including: (E–H) Microscopic imaging of Giemsa-stained cells on the basal side of the trans well insert. (I–L) Image J quantitation of Giemsa-stained cells on the basal side of the trans well. (M–P) Hemocytometer-based enumeration of basal chamber cells. (Q–T) Spectrophotometric absorbance-based quantitation of basal chamber cells. For quantitative analysis of migration events, cells from five different fields were counted and then averaged. Error bars represent standard deviations. Significance was taken at *P* < 0.05^*^, *P* < 0.01^**^, and *P* < 0.001^***^. ns = not significant. Experiments were repeated more than three times with similar results.

Since the function of BST-2 on virus inhibition requires wild type BST-2 with functional ectodomain (ECD) and cytoplasmic tail (CT), we hypothesized that these BST-2 domains may play a role in BST-2-mediated regulation of cell motility. To test this hypothesis, we performed wound healing and trans well migration experiments using our previously described BST-2-suppressed 4T1 series overexpressing variants of BST-2 [9], including: wild type BST-2 that is predominantly expressed as dimers (designated OE BST-2D), dimerization-deficient BST-2 that is predominantly expressed as monomers (designated OE BST-2M), and dimerization-proficient, signaling-deficient BST-2 in which the cytoplasmic tail tyrosine residues at positions 6 and 8 had been substituted with alanine residues (designated OE BST-2DΔTy). As expected, OE BST-2D overexpression completely rescues wound closure, while OE BST-2M cells had modest effect on wound closure (Figure 4A). In contrast, OE BST-2DΔTy failed to rescue wound closure (Figure 4A). Further, wound closure assay was used in a competitive co-culture experiment to determine if OE BST-2D will rescue migration of OE BST-2DΔTy cells. As shown in Figure 4B, OE BST-2D cells maintain superior migratory ability compared to OE BST-2DΔTy cells and fail to endow OE BST-2DΔTy cells migration potential (Figure 4B). Comparison of the rate of wound closure show a decrease in wound area of mono-cultured cells at 24 hours calculated as relative wound area of 84%, 41%, 67%, and 84% in vector, OE BST-2D, OE BST-2M, and OE BST-2DΔTy expressing cells respectively (Figure 4C). A similar decrease in rate of migration was observed in competitive co-culture assay where expression of OE BST-2D but not OE BST-2DΔTy cells increases relative fluorescence and cell numbers in wound area (Figure 4D). Similar to wound healing, trans migration experiments show that in four isogenic cancer cell lines―4T1, 4T07, 168FARN, and 67NR, restoration of BST-2 expression in shBST-2 cells rescues cell migration, as documented by microscopy (Figure 4E–4H), image J analysis of microcopy data (Figure 4I–4L), hemocytometer-based cell counting (Figure 4M–4P), and spectrophotometry-based absorbance (Figure 4Q–4T). However, substitution of the cytoplasmic tail tyrosine with alanine residues did not rescue migration in any of the isogenic cell lines using any of the assays (Figure 4E–4T).

### BST-2 cytoplasmic tail regulates breast cancer cell invasion

**Figure 5:**
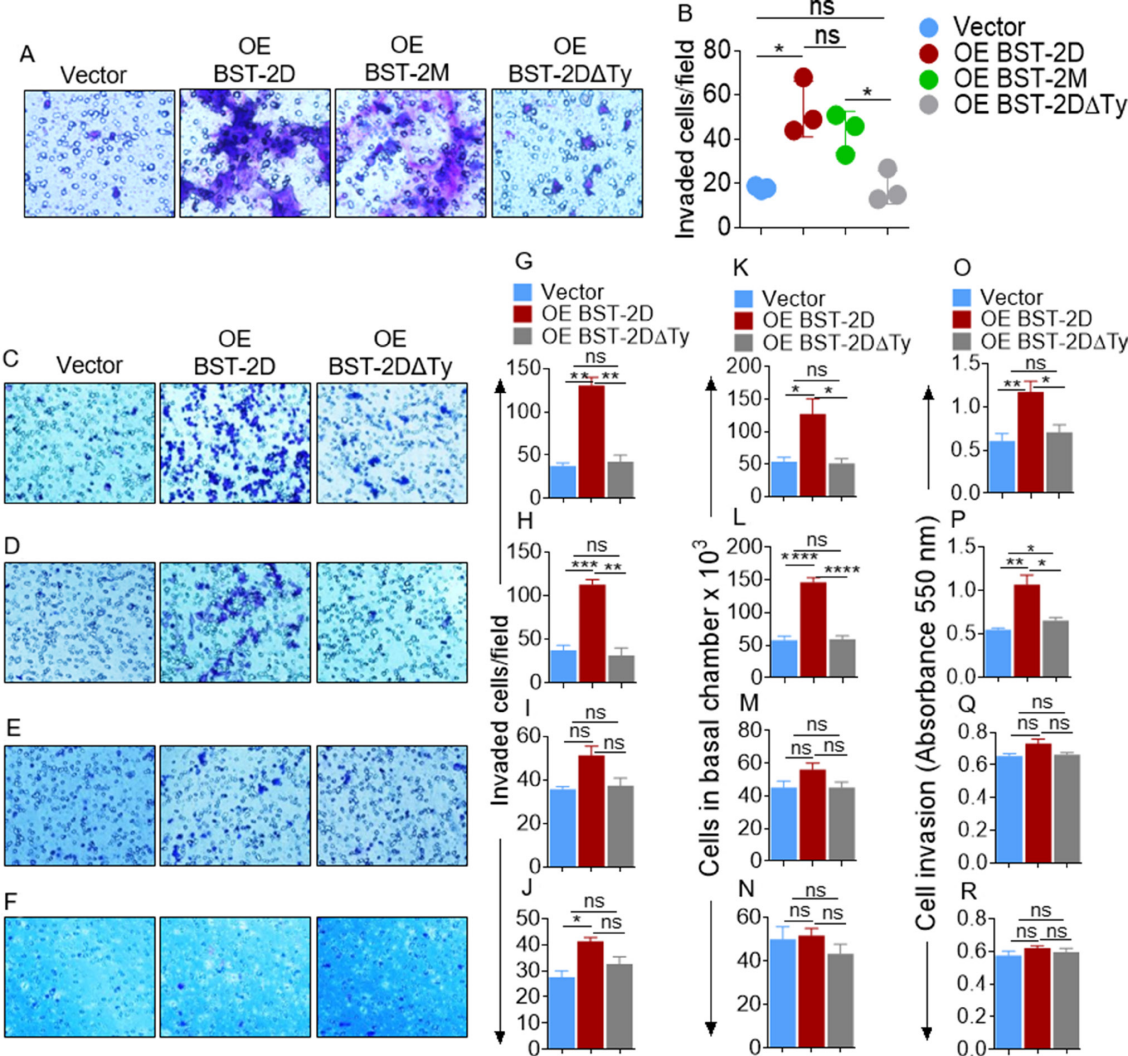
Identification of the domain of BST-2 that controls cell invasion (**A**–**B**) Representative images and quantification of invasion of MCF-7 cells expressing variants of BST-2 (shBST-2 (vector), OE BST-2D, OE BST-2M or OE BST-2DΔTy). (**C**–**F**) Microscopic images of Giemsa-stained invaded isogenic 4T1, 4T07, 168FARN, 67NR shBST-2 cells expressing variants of BST-2 (shBST-2 (vector), OE BST-2D, OE BST-2M or OE BST-2DΔTy). (G–J) Image J quantitation of Giemsa-stained invasive cells. (K–N) Number of invaded cells enumerated using hemocytometer. (O–R) Quantitation of invaded cells by spectrophotometer measurement of absorbance. Cells from five different fields were analyzed. Error bars represent standard deviations. Significance was taken at *P* < 0.05^*^, *P* < 0.01^**^, *P* < 0.001^***^, and *P* < 0.0001^****^. ns = not significant. Experiments were repeated more than three times with similar results.

Analyses of *in vitro* invasion of breast cancer cells produced a similar result as migration. Specifically, the moderately invasive MCF-7 cells expressing OE BST-2DΔTy are non-invasive, while OE BST-2M rescues invasion by 57% and OE BST-2D rescues cell invasion by 169% (Figure 5A–5B). The significance of the tyrosine residues in the BST-2 cytoplasmic tail was further demonstrated in cells with varied metastatic potential using various parallel assays. Overexpression of BST-2D rescues invasion of the highly metastatic 4T1 cells (Figure 5C, 5G, 5K, 5O) and potentiates invasion of the moderately metastatic murine 4TO7 cells (Figure 5D, 5H, 5L, 5P). However, overexpressing BST-2D does not change invasion of the weakly metastatic 168FARN (Figure 5E, 5I, 5M, 5Q) or the invasion of the non-metastatic 67NR cells (Figure 5F, 5J, 5N, 5R). As expected, BST-2DΔTy overexpression was unable to rescue invasion in these cells (Figure 5C–5R). These experiments reveal that the BST-2 extracellular domain does not play a key role in cell motility; but we identify the BST-2 cytoplasmic tail YxY motif as indispensable in the invasion of aggressive breast cancer cell lines.

### The tyrosine residues in the cytoplasmic tail of BST-2 regulates cluster-based cell invasion

**Figure 6:**
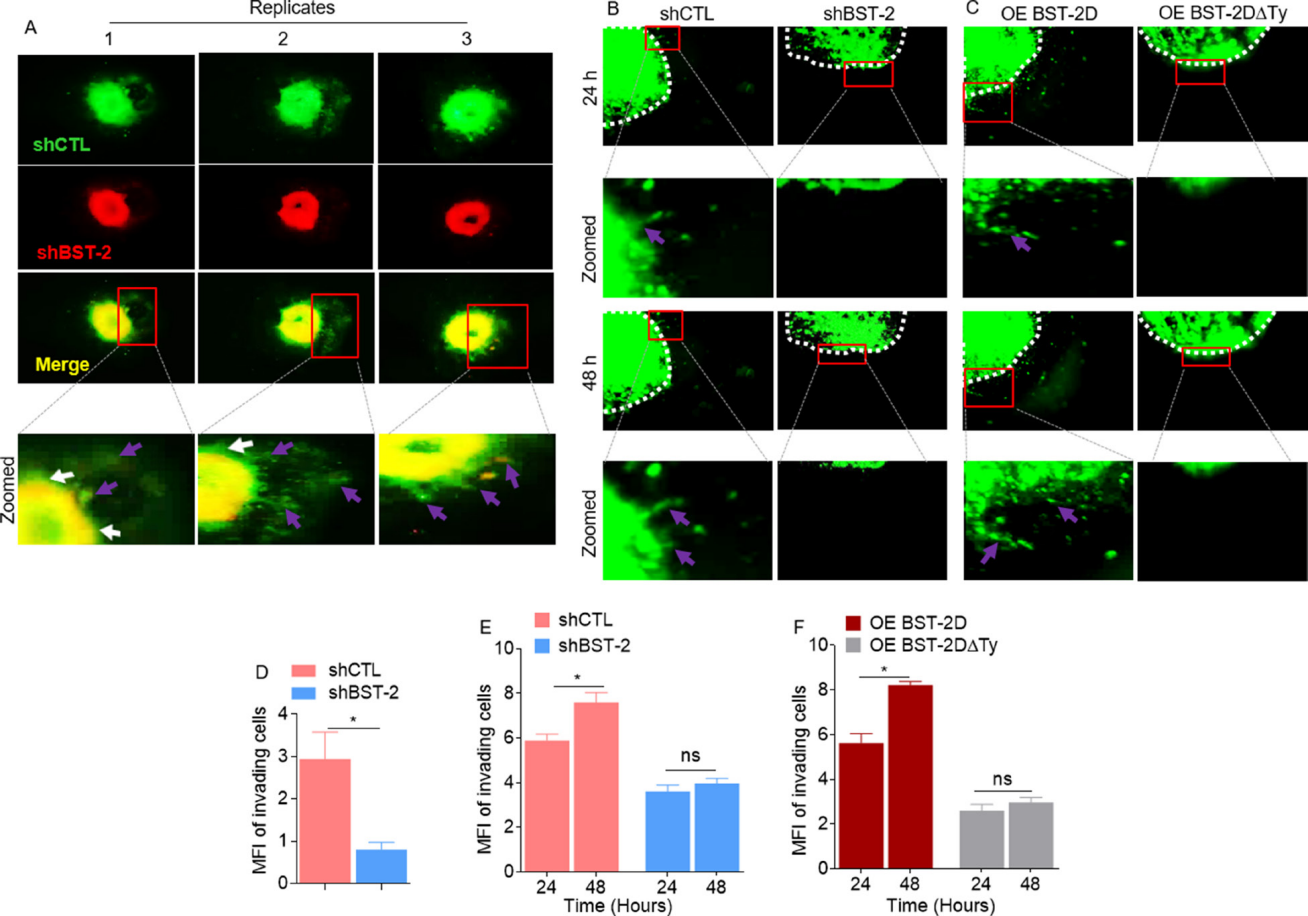
Role of BST-2 in collective cancer cell invasion (**A**–**C**) Representative images of co-culture spheroids of shCTL (green) and shBST-2 (red), mono-culture spheroids of 4T1 shCTL and shBST-2, and mono-culture spheroids of 4T1 cells expressing BST-2 dimers (OE BST-2D) or a mutant BST-2 (OE BST-2DΔTy) embedded in Matrigel for the indicated times. Red boxes depict increasing invasive structures. White arrow heads indicate expanding spheroid boundaries while purple arrows show collective invasive structures. White dotted lines highlight the spheroid boundaries. Images are zoomed in rows 2 and 4. (**D**–**F**) Quantitation of mean fluorescence intensity (MFI) of co-cultured shCTL and shBST-2, mono-cultured shCTL and shBST-2, and mono-cultured OE BST-2D and OE BST-2DΔTy invasive strands respectively. Error bars represent SEM. Significance was taken at *P* < 0.05^*^. Experiments were repeated more than three times with similar results.

Thus far, our data have shown that BST-2 promotes both protease-independent and protease-dependent cell motility. A critical factor influencing cell invasion is the physio-structural constraints mounted by the extracellular matrix. Consequently, we used a 3D spheroid system to examine the role of BST-2 in overcoming such structural constrain during invasion. Co-culture multicellular spheroids were made by mixing equal numbers of PKH67Green-labeled shCTL with PKH26Red-labeled shBST-2 cells. Using this system, we showed that BST-2-expressing cells sent out collective, bud-like protrusions into the matrix (Figure 6A, red boxes and arrow heads in enlarged images). In contrast, when BST-2 is suppressed, cells did not productively invade the matrix and collective invasion was diminished (Figure 6A), indicating that the extent of collective cell invasion is dependent on BST-2. A similar observation was made when mono-culture spheroids were formed with shCTL or shBST-2 cells—where cells expressing high BST-2 invaded Matrigel collectively while cells expressing low BST-2 did not (Figure 6B, red boxes and arrow heads in enlarged images). As expected, OE BST-2D rescues collective invasion in shBST-2-suppressed cells while OE BST-2DΔTy was unable to rescue invasion (Figure 6C, red boxes and arrow heads in enlarged images). Measurement of the invasive structures shows a progressive increase in fluorescent intensity in cells that express high levels of BST-2 (Figure 6D–6F, compare pink and blue bars; red and gray bars). By 48 h of culture, cells collectively advance through matrix substrate as indicated by increased invasive strands (Figure 6B) and increased florescence (Figure 6E), which was dependent on the variant of BST-2 in the cells (Figure 6C and 6F). Together, these data reveal that BST-2 controls collective cell motility through physiologically three-dimensional matrices and that the tyrosine residues in the BST-2 cytoplasmic tail play a crucial role in this process.

## DISCUSSION

Although BST-2 has been linked to breast cancer cell migration and invasion [6, 26], in this study, we demonstrate that BST-2 overexpression is a requirement for enhanced migratory response in various cancer cells. We also systemically used structure-function analysis of the BST-2 protein to identify the domain of BST-2 involved in proteolytic and non-proteolytic motility of breast cancer cells. The BST-2 extracellular domain was found to modestly contribute to cell migration and invasion. However, both non-proteolytic (migration) and proteolytic (invasion) functions of BST-2 are dependent on the BST-2 cytoplasmic tail. Specifically, substitution of the cytoplasmic tail tyrosine residues abrogates BST-2-mediated enhancement of cell migration and invasion. The intracellular pathways controlled by the BST-2 cytoplasmic tail in the context of cancer cell migration and invasion are unknown. However, we have previously shown that BST-2 is activated via cancer cell to cancer cell interactions [9], resulting in phosphorylation of the double tyrosine motif on the BST-2 cytoplasmic tail [9]. It is therefore possible that activation of BST-2 may lead to the activation of NF-kB and possibly subsequent induction of the expression of genes such as MMP-1 [27], MMP-9 [28], COX-2 [29], VEGF [30] involved in cell migration and invasion.

It is known that at least two stimuli are involved in cancer cell motility. A faster motility induced by soluble factors and a much slower basal motility orchestrated by adhesion receptors and other factors [31]. Intra- and extra- cellular factors that regulate cancer cell motility have been demonstrated and they include growth factors [32, 33], chemokines [34], proteases [35], and extracellular matrix proteins. Our finding that suppression of BST-2 impairs cell motility and that overexpression of exogenous BST-2 rescues migration and invasion of BST-2-suppressed cells argues for a role for BST-2-mediated intracellular regulators of cell motility. Although we cannot rule out the involvement of extracellular factors in BST-2-regulated cell motility, co-culture spheroid study indicates that BST-2-sufficient cells are unable to efficiently promote motility of BST-2-deficient cells.

In our study, we show that cells expressing BST-2 and in particular, the tyrosine residues in the cytoplasmic tail promotes cohesive strand-cell invasion in a spheroid model of cell invasion. Results of our trans well-based or Boyden chamber invasion assays along with the 3D spheroid assay suggest that BST-2 may promote individualized as well as collective invasion. The notion of BST-2-mediated collective cell invasion is intriguing. It is however unclear how BST-2 endows a group of adherent epithelial cancer cells motile invasive behavior for collectivity.

In a previous study, we showed that BST-2 expression promotes adhesive interaction between cells and between cells and ECM substrates [6]. Recently, we demonstrated that it is the variant and not the level of BST-2 in cancer cells that regulates cancer cell clustering, which in turn promotes anoikis resistance [9]. Cell-ECM adhesion plays critical roles during cancer metastasis in part by anchoring malignant spheroids to the ECM of the basement membrane and target organs. It is therefore plausible that cancer cells accomplish degradation of the basement membrane and collective invasion through BST-2-mediated adhesive interactions, cancer cell signaling, and extracellular signaling cues among functionally similar and distinct cancer and stromal cells that express BST-2. This BST-2-directed role may be achieved in part through the activities of the tyrosine residues located in the cytoplasmic domain of BST-2, that have been implicated in BST-2-mediated signal activation [13, 36, 37].

**Figure 7:**
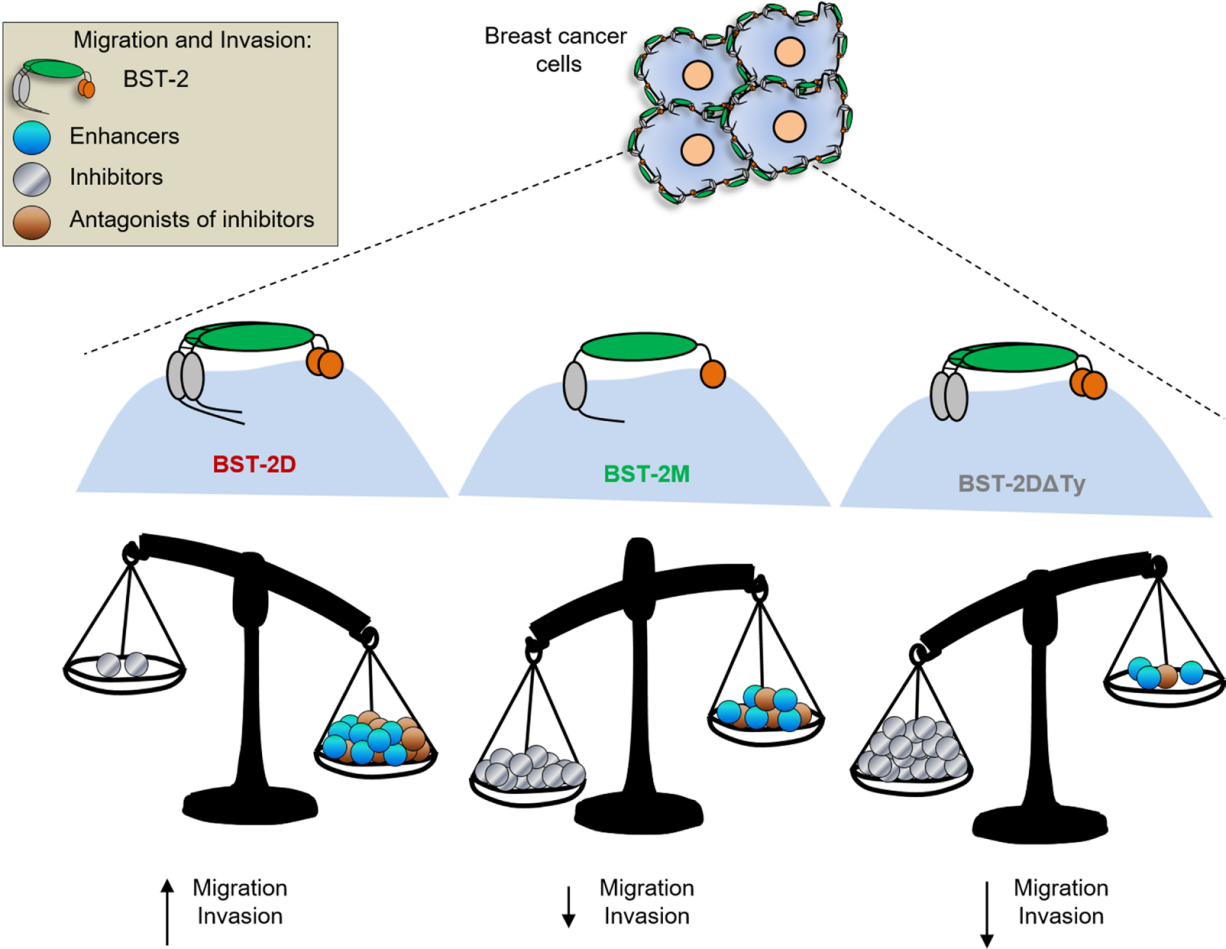
Model of BST-2-induced cell motility: BST-2 in breast cancer cells initiate intracellular events that enhance cancer cell migration and invasion We propose a model in which BST-2- promote the expression of i) factors that promote cancer cell motility (enhancers), ii) factors that inhibit cell motility (inhibitors), or factors that inhibit the activity or expression of motility enhancers (antagonists of inhibitors). In our study, expression of BST-2DΔTy in cancer cells reduced migration of cancer cells suggesting that the YXY motif on the cytoplasmic tail of BST-2 may regulate the expression and activities of motility inhibitors.

The reason for the differences in motility besides BST-2 are unknown; but likely due to differences in the cell-cell and cell-matrix interactions that may result in invasion promoting signal transduction. Whether BST-2 expressing cancer cells use membrane-anchored proteinases to remodel the basement membrane and to migrate through cross-linked ECM substrates remains to be determined. However, it is clear from our mutational and functional studies that potent anti-motility factor(s) may be present in cells lacking the cytoplasmic tail tyrosine residues (Figure 7).

Therefore, future studies should identify the BST-2-directed cancer cell program that regulates stromal network structure and promotes individualized and collective cancer cell invasion. Interrogation of the molecular signature of cells expressing variants of BST-2 may provide relevant clues as to the mechanisms of BST-2-mediated regulation of cell motility. This knowledge will facilitate targeting BST-2-dependent pro-motility pathways; a move that will provide avenues to explore the use of anti-motility signals on tumors.

## MATERIALS AND METHODS

### Cell lines and Media

The murine triple negative breast cancer cell line 4T1 was a kind gift from Dr. Lyse Norian of the University of Iowa. MCF-7 cells (luminal A human breast cancer cell line) and MDA-MB-231 cells (triple negative human breast cancer cell line) were kindly provided by Dr. Weizhou Zhang of the University of Iowa. 4TO7, 168FARN, and 67NR were kindly provided by Dr. Jing Yang of University California, San Diego. TZM-bl, SUPT1, and U937 cells were obtained from NIH NIAID AIDS reagents program. All cells were maintained in their respective complete medium according to ATCC and NIH NIAID AIDS reagents guidelines. Complete medium used in culturing cells contains 10% fetal bovine serum (FBS). Media used in basal chamber of trans well inserts for migration and invasion experiments contain 30% FBS and 5 μg/ml fibronectin. Cell starvation was performed by culturing cells in medium without FBS.

### Suppression of BST-2 expression in murine isogenic cell lines

4T1 shBST-2 cells in which endogenous mouse BST-2 was shRNA-suppressed and 4T1 shControl (shCTL) cells have been previously described [6]. To stably knock down BST-2 in 4TO7, 168FARN, and 67NR cells, we used the protocol described previously for 4T1 cells [6].

### Generation of BST-2-suppressed human cell lines

MDA-MB-231 cells were transfected with a scramble shRNA (shControl, (shCTL)) or shRNAs targeting human BST-2 (shBST-2-h1 and shBST-2-h2). These constructs were purchased from GeneCopoeia; shCTL (Cat # CSHCTR001-LvmH1), shBST-2-h1 (Cat # HSH017844-1-lVMh1) and shBST-2-h2 (Cat # HSH017844-4-LvmH1). shRNA constructs were transfected using Lipofectamine 2000 (Life Technologies) according to the manufacturer's instructions. Transfected cells were selected with Puromycin at 2.5 μg/ml and stable cells were used in all experiments. In addition, TZM-bl, SUP-T1, U937 cells were transfected with shCTL and shBST-2-h2 as described for MDA-MB-231 cells.

### Generation of BST-2-overexpressing cancer cells

To generate cells expressing different BST-2 variants, shBST-2-expressing 4T1 cells were stably transfected (over expressed, OE) with expression constructs for signaling and dimerization-competent wild type human BST-2 (BST-2 dimer, OE BST-2D), dimerization-mutant BST-2 in which cysteine residues at positions 53, 63 and 91 were replaced with alanine residues, (BST-2 monomers, OE BST-2M), or dimerization-competent, signaling-impaired BST-2 in which tyrosine residues at positions 6 and 8 were replaced with alanine residues, (tyrosine mutant, OE BST-2DΔTy), or empty pcDNA3.1 control (vector). These 4T1 cell line series have been previously described [9]. Note that there is about 36% homology between murine and human BST-2 and that the murine shRNA sequences which targets murine BST-2 has no effect on human BST-2 constructs.

### Assessment of BST-2 protein and mRNA levels

Western blots were performed as previously described [38–40]. Similarly, isolation of RNA, reverse transcriptase PCR (RT-PCR), and reverse transcriptase quantitative PCR (RT-qPCR) were accomplished as previously described [41–48]. Primer sequences are as follows: GAPDH-Forward: 5′-CCCCTTCATTGACCTCAACTACA-3′, Reverse: 5′-CGCTCCTGGAGGATGGTGAT-3′; mouse BST-2 (mBST-2)-Forward: 5’-TCAGGAGTCCCTGGAGAAGA -3’, Reverse: 5’-ATGGAGCTGCCAGAGTTCAC-3’; and human BST-2 (hBST-2)-Forward: 5’-AGAAG GGCTTTCAGGATGTG-3’ Reverse: 5’-CTTTTGTC CTTG GGCCTTCT-3’.

### Migration (wound healing) assay

Cells of interest were seeded in 24-well plates to form perfect confluent monolayer as previously described [6, 9]. The monolayers were scratched using pipette tip. Fresh medium was added to the wells and cells were allowed to migrate for the indicated times. Cells were imaged with Nikon Eclipse Ti microscope adjusted with a Nikon digital sight camera. Images were processed and rate of wound closure blindly analyzed using Image J software. In some experiments, the cells were labelled with PKH dye as previously described [6, 9, 41, 42]. Plates were taken to the Olympus iX80 inverted microscope with temperature regulated chamber and set for imaging for 0 and 24 h. Fluorescence was measured from the area inside of the wound at 0 and 24 h time points. Rate of migration was calculated as relative 24 h fluorescence intensity normalized to the 0 h time point. In parallel, the number of cells inside the wound area were blind-counted at 0 and 24 h. Values for the 24 h cell numbers were normalized to the 0 h time point.

### Trans well migration (Boyden chamber) assay

The apical chamber of 24-well cell culture inserts (Millipore) were seeded with equivalent numbers (250,000) of previously starved cells of interest in serum-free medium as previously described [6, 9]. Culture medium containing 30% FBS and 5 μg/ml fibronectin was added to the basal chamber of the unit. Cells were allowed to migrate through the membranous barrier for 22 hours at 37°C. Following incubation, cells that did not migrate were removed from the apical side of the filter by wiping with PBS-wetted Kim wipe, followed by PBS rinse to remove remaining cell debris. The filters containing migrated cells were fixed with 4% paraformaldehyde (PFA) for 5 minutes, washed twice with 1x PBS, permeabilized with 100% methanol for 25 minutes, labeled with Giemsa stain (for 15 minutes at room temperature), and imaged using a Nikon Eclipse Ti microscope adjusted with a Nikon digital sight camera. Images were processed using Image J software. Cells from 3 to 5 different fields were blind-counted and averaged. In parallel, cells that migrated to the basal chamber were enumerated using the hemocytometer cell counting method. Number of migrated cells was calculated as:

$$\begin{array}{l} \text{Total number of migrated cells}=\\ (\text{total cells counted }\times \frac{\text{dilution factor}}{\#\text{of squares counted}}\times 10^{4})\;\times \text{original based chamber volume} \end{array}$$

In parallel, basal chamber cells were collected, number enumerated and presented as cells in basal chamber ×10^3^. In addition, basal chamber cells were seeded in a 96 well plate for 5 hours, medium removed and cells stained with 0.5% crystal violet for 30 min. After washing with 1X PBS twice, cells were treated with 0.2 % SDS for 10–15 min. Absorbance was read with a Tecan microplate reader at 550 nm. Spectrophotometric absorbance correlates with cell migration, where increased absorbance correlates with higher cell motility [49].

### Invasion assay

The apical chamber of 24-well cell culture inserts (Millipore) were coated with 1.5 mg/ml of Matrigel (100 μl) (Sigma-Aldrich) and allowed to solidify for 3 hours as previously described [6]. A total of 250,000 previously starved cells of interest expressing various forms of BST-2 were suspended in serum-free medium and plated on top of the Matrigel layer. 600 μl of culture medium containing 30% FBS and 5 μg/ml fibronectin (Sigma-Aldrich) was added to the basal chamber of the unit. Cells were allowed to invade through the membranous barrier for 24 hours at 37°C. Noninvasive cells were washed off; invasive cells were fixed with 4% PFA, permeabilized with 100% methanol, labeled with Giemsa stain and imaged as described in the previous paragraph. Images were processed using Image J software. Cells from five different fields were blind-counted and averaged as depicted in the equation below.

$$\begin{array}{l} \text{Total number of invaded cells}=\\ (\text{total cells counted }\times \frac{\text{dilution factor}}{\#\text{of squares counted}}\times 10^{4})\;\times \text{original based chamber volume} \end{array}$$

Additionally, invasion rate was determined by determining the total number of invaded cells in the basal chamber either by counting or spectrophotometric absorbance as described for migration.

### 3D invasion assay

4T1 cells expressing variants of BST-2 were grown to 80% confluency and starved for 3 hours with serum free RPMI. After starvation, cells were trypsinized and centrifuged to pellet. After centrifugation, fluid was drained off and pellets were labeled with PKH67- dye (Sigma). Equivalent numbers of relevant 4T1 cells labelled with PKH67Green were counted and re-suspended in 1.5 mg/ml of Matrigel as 20,000 cells/100 μl of 1.5 mg Matrigel and added to Poly-Hema coated 96 well plate. Cells were quickly centrifuged for 15 min at 500 Xg (centrifuge was pre-cooled at 4°C, to prevent Matrigel polymerization). After centrifugation, plate was placed in incubator at 37°C with 5% CO_2_ for 3 hours, followed by addition of media with 30% FBS on top of the Matrigel. Plate was taken to the Olympus iX80 inverted microscope with temperature regulated chamber and set for imaging up to 48 hours. The image J software was used to measure the mean fluorescence intensity of invading cells.

For 3D co-culture invasion, 4T1 shCTL cells were labelled with to PKH67Green dye and shBST-2 cells were labelled with PKH26Red. Equivalent numbers of the cells were mixed together and re-suspended in 1.5 mg of Matrigel. 1:1 cell mixtures were plated on Poly-Hema coated 96 well-plate and invasion analyzed as describe in the paragraph above.

### Statistics

Statistical analysis was performed with unpaired *t* test assuming Gaussian distribution with Welch's correction (GraphPad Prism software). Error bars represent standard deviation for transcript data and standard error of the mean (SEM) for other data. A probability (P) value of 0.05 or lower was considered significant.

## CONCLUSIONS

We have identified the structural requirements for BST-2-regulated migration and invasion of breast cancer cells. Identification of the pro-motility factors present in cells with elevated BST-2 as well as the anti-motility factors in cells with reduced BST-2 or cells bearing mutations in the BST-2 cytoplasmic tail is crucial. The anti-motility factors when identified and validated in preclinical models of breast cancer could act as motility “stop” signals for migration and invasion of tumor cells. Given the broad functionality of BST-2 and its ability to regulate signals involved in cancer, we expect that the pro-motility and anti-motility factors may be part of the global cancer interactome.

## SUPPLEMENTARY MATERIALS FIGURE



## Abbreviations

β-ME: β-Mercaptoethanol
BrdU: Bromodeoxyuridine or 5-bromo-2’-deoxyuridine
BST-2: Bone marrow stromal antigen 2
CT: Cytoplasmic tail
ECD: Extracellular domain
ECM: Extracellular matrix
FBS: Fetal bovine serum
FN: Fibronectin
MTT: 3-(4,5-Dimethylthiazol-2-yl)-2,5-Diphenyltetrazolium Bromide
PBS: Phosphate-Buffered Saline
PFA: Paraformaldehyde
OE: Over expressed
OE BST-2D: BST-2 dimers
OE BST-2M: BST-2 monomers
OE BST-2DTy: BST-2 dimers impaired for signaling
