# Supplementary material for: Structural determinant of BST-2-mediated regulation of breast cancer cell motility: a role for cytoplasmic tail tyrosine residues
Source: Oncotarget. 2017 Nov 29;8(66):110221–33. doi: 10.18632/oncotarget.22753 (PMC5746378; doi:10.18632/oncotarget.22753)
Supplement: Supplementary file 1 [file oncotarget-08-110221-s001.pdf]

Structural determinant of BST-2-mediated regulation of breast cancer cell motility: a role for cytoplasmic tail tyrosine residues

SUPPLEMENTARY MATERIALS

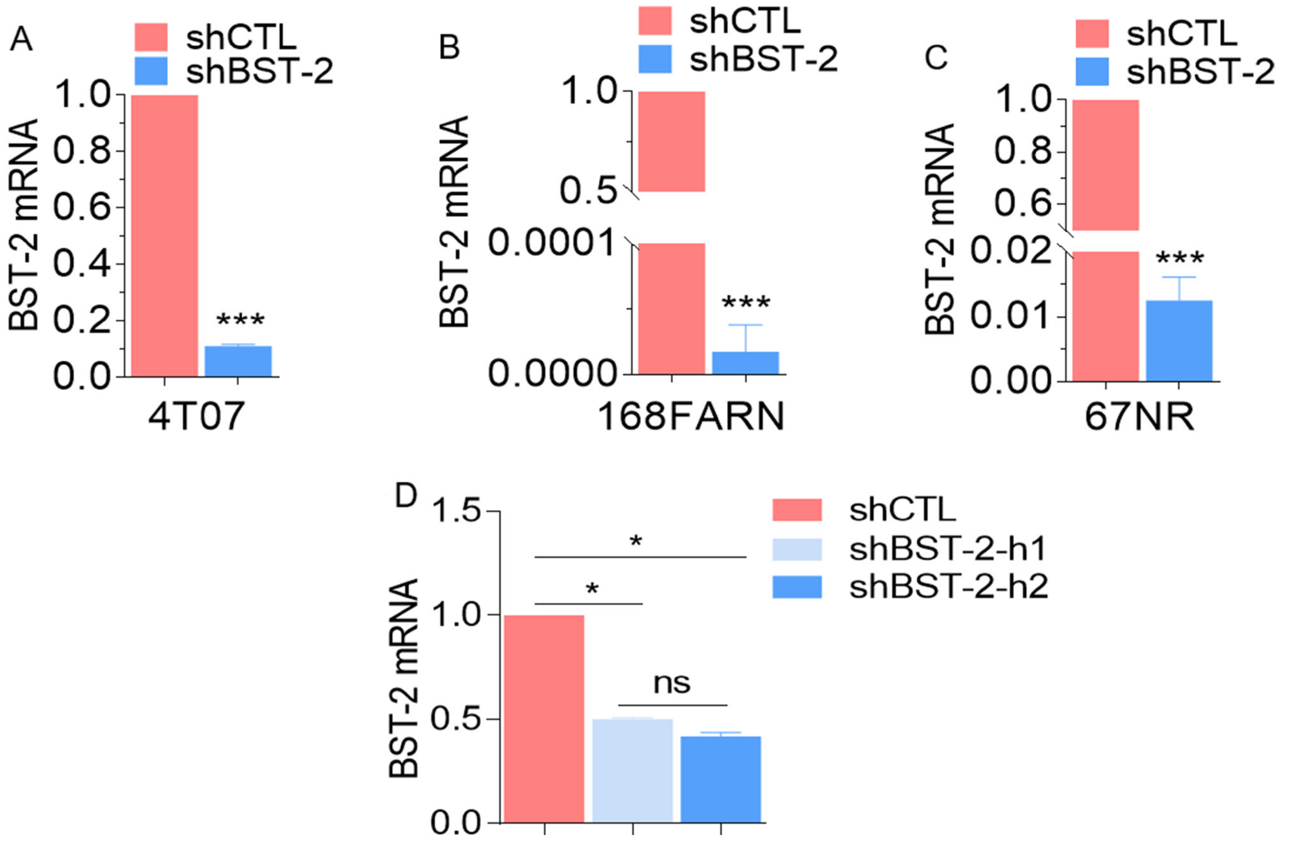

Supplementary Figure 1: Generation of BST-2-suppressed cells. (**A–C**) Relative levels of murine BST-2 mRNA in murine cells stably expressing a scramble shRNA (shCTL) or BST-2-targeting shRNA. (**D**) mRNA levels of BST-2 in MDA-MB-231 cells stably expressing a scramble shRNA (shCTL) or two different BST-2-targeting shRNAs (shBST-2-h1, shBST-2-h2). RT-qPCR data were normalized to GAPDH.
